# The Effect of Tuberculosis Antimicrobials on the Immunometabolic Profiles of Primary Human Macrophages Stimulated with *Mycobacterium tuberculosis*

**DOI:** 10.3390/ijms222212189

**Published:** 2021-11-10

**Authors:** Christina Cahill, Dónal J. Cox, Fiona O’Connell, Sharee A. Basdeo, Karl M. Gogan, Cilian Ó’Maoldomhnaigh, Jacintha O’Sullivan, Joseph Keane, James J. Phelan

**Affiliations:** 1 TB Immunology Group, Department of Clinical Medicine, Trinity Translational Medicine Institute, Trinity College, Dublin, Ireland; cahillch@tcd.ie (C.C.); DOCOX@tcd.ie (D.J.C.); basdeos@tcd.ie (S.A.B.); gogank@tcd.ie (K.M.G.); cilianom@yahoo.ie (C.Ó.); josephmk@tcd.ie (J.K.); 2 Department of Surgery, Trinity Translational Medicine Institute, Trinity College Dublin, St James’s Hospital, Dublin 8, Ireland; oconnefi@tcd.ie (F.O.); osullij4@tcd.ie (J.O.)

**Keywords:** tuberculosis, drug-resistant tuberculosis, host-directed therapy, antimicrobials, glycolysis, oxidative phosphorylation, mitochondrial function, lipopolysaccharide, bioenergetics

## Abstract

Tuberculosis (TB) remains a global health challenge. Patients with drug-sensitive and drug-resistant TB undergo long, arduous, and complex treatment regimens, often involving multiple antimicrobials. While these drugs were initially implemented based on their bactericidal effects, some studies show that TB antimicrobials can also directly affect cells of the immune system, altering their immune function. As use of these antimicrobials has been the mainstay of TB therapy for over fifty years now, it is more important than ever to understand how these antimicrobials affect key pathways of the immune system. One such central pathway, which underpins the immune response to a variety of infections, is immunometabolism, namely glycolysis and oxidative phosphorylation (OXPHOS). We hypothesise that in addition to their direct bactericidal effect on *Mycobacterium tuberculosis* (Mtb), current TB antimicrobials can modulate immunometabolic profiles and alter mitochondrial function in primary human macrophages. Human monocyte-derived macrophages (hMDMs) were differentiated from PBMCs isolated from healthy blood donors, and treated with four first-line and six second-line TB antimicrobials three hours post stimulation with either iH37Rv-Mtb or lipopolysaccharide (LPS). 24 h post stimulation, baseline metabolism and mitochondrial function were determined using the Seahorse Extracellular Flux Analyser. The effect of these antimicrobials on cytokine and chemokine production was also assayed using Meso Scale Discovery Multi-Array technology. We show that some of the TB antimicrobials tested can significantly alter OXPHOS and glycolysis in uninfected, iH37Rv-Mtb, and LPS-stimulated hMDMs. We also demonstrate how these antimicrobial-induced immunometabolic effects are linked with alterations in mitochondrial function. Our results show that TB antimicrobials, specifically clofazimine, can modify host immunometabolism and mitochondrial function. Moreover, clofazimine significantly increased the production of IL-6 in human macrophages that were stimulated with iH37Rv-Mtb. This provides further insight into the use of some of these TB antimicrobials as potential host-directed therapies in patients with early and active disease, which could help to inform TB treatment strategies in the future.

## 1. Introduction

The use of antimicrobials for the treatment of tuberculosis (TB) has been the mainstay for almost 50 years now. Although the eradication of TB in patients with drug-sensitive TB is quite efficacious, treatment for patients with drug-resistant TB involves a much longer, gruelling, and complex drug regimen, often involving the administration of numerous toxic antimicrobials. This necessitates the need to identify therapies to help to improve the current state-of-the-art treatments. Recently, a significant amount of attention has turned to the use of host-directed therapies to modulate immune responses in infected host immune cells [1]. These strategies have been hypothesized to work through augmenting anti-inflammatory [2,3], pro-inflammatory [2,4,5,6,7], and immunometabolic processes [4,6,7,8]. Interestingly, in addition to their bactericidal effects, some studies now suggest that TB antimicrobials could also help during TB disease through the modulation of host immune responses by host-directed strategies [9]. In addressing this question, the literature highlights some contradictory findings across different cell types and gives us some insight into how TB antimicrobials could affect a variety of cellular processes, while highlighting the need for more studies that specifically detail and characterise these immunomodulatory effects, particularly in primary human cell models. 

How TB antimicrobials affect proinflammatory, anti-inflammatory, and immunometabolic profiles of infected hosts highlights a significant research gap in the field. For example, even though it is now well known that the metabolic state of human macrophages underpins their response to *Mycobacterium tuberculosis* (Mtb) infection [10,11], how TB antimicrobials directly affect glycolytic and oxidative phosphorylation (OXPHOS) profiles in human macrophages needs to be recognized. Moreover, characterising the effect of TB antimicrobials on innate immune cell function could offer immediate therapeutic effects. For example, while considering the resistance of a particular Mtb strain, administering a TB patient suffering from an adverse inflammatory response with a TB drug that elicits anti-inflammatory properties, instead of a drug known to promote pro-inflammatory or oxidative processes, could aid in the treatment process and offer alternative treatment modalities. This could also conceivably consist of administering a patient with drug-resistant TB, who is on an array of second line drugs, with a first line drug known to elicit anti-inflammatory or pro-autophagic properties, even if the patient is also resistant to the first line drug. Thus, uncovering additional benefits to using these antimicrobials more optimally could also help to alleviate treatment times, lower costs, and inform future studies.

In the current study, we examined if four first-line and six second-line TB antimicrobials (outlined in Table 1) can modulate immunometabolic profiles and alter mitochondrial function in primary human monocyte-derived macrophages (hMDMs) stimulated with iH37Rv-Mtb and lipopolysaccharide (LPS). We show that TB antimicrobials differentially alter both OXPHOS and glycolysis in unstimulated, iH37Rv-Mtb and LPS-stimulated hMDMs, with some of these observations linked with alterations in mitochondrial function. Our results reveal that that TB antimicrobials do not affect cytokine or chemokine levels in hMDMs stimulated with iH37Rv-Mtb, but significantly alter levels in LPS-stimulated hMDMs, also suggesting an application for these drugs in the treatment of gram-negative bacteria. Our data may uncover a use for these antimicrobials in non-TB patients with resistant, difficult to treat infectious diseases. Importantly, this study provides some insight into the use of TB antimicrobials in TB patients with active disease which could help to inform TB treatment strategies in the future.

## 2. Results

### 2.1. TB Antimicrobials Differentially Affect Glycolysis and Oxidative Phosphorylation Levels in Unstimulated, iH37Rv-Mtb- and LPS-Stimulated hMDMs

TB antimicrobials optimally elicit direct bactericidal effects on mycobacteria, both in cellular and axenic models of TB infection [5,22,23,24,25,26,27]. Accordingly, examining how these antimicrobials affect immunometabolic profiles and mitochondrial function in parallel could offer some insight into how these antimicrobials potentially affect immune function [7], particularly in patients with active TB who are subjected to long-term antimicrobial treatment regimens. Moreover, recent studies have started to identify potential adjunctive host-directed therapies that aim to augment cellular bioenergetics to support immune function during the early stages of Mtb infection [6,28]. Therefore, a more thorough understanding of the immunometabolic capabilities of current TB antimicrobials could help to inform and tailor more personalised future TB treatment strategies.

**Figure 1:**
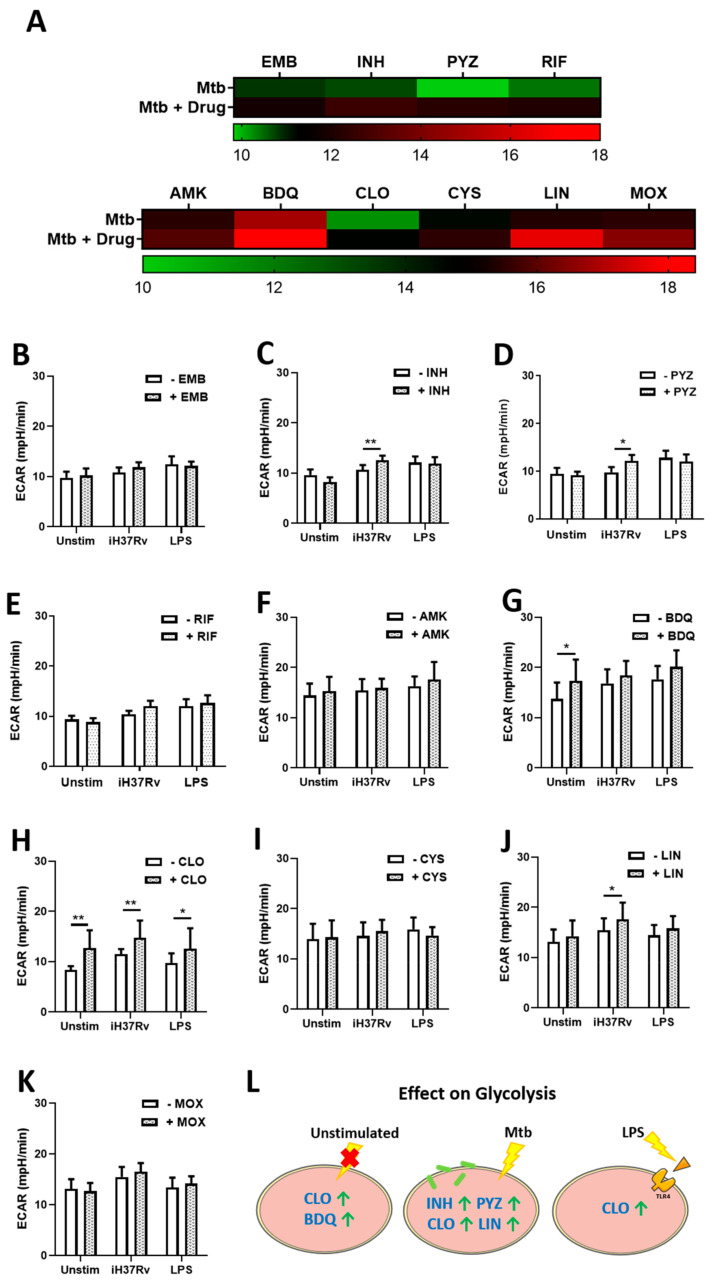
Isoniazid, pyrazinamide, bedaquiline, clofazimine and linezolid increase glycolytic profiles in unstimulated, iH37Rv-Mtb-stimulated and LPS-stimulated hMDMs. (**A**) Illustrative heat map showing changes in ECAR in response to treatment with first-line (top) and second-line (bottom) TB antimicrobials. hMDMs, differentiated from PBMCs isolated from healthy blood donors, were treated with (**B**) ethambutol (5 µg/mL), (**C**) isoniazid (1 µg/mL), (**D**) pyrazinamide (2 µg/mL), (**E**) rifampicin (2 µg/mL), (**F**) amikacin (5 µg/mL), (**G**) bedaquiline (5 µg/mL), (**H**) clofazimine (2 µg/mL), (**I**) cycloserine (5 µg/mL), (**J**) linezolid (15 µg/mL) or (**K**) moxifloxacin (12.5 µg/mL) three hours post stimulation with either iH37Rv-Mtb or LPS (100 ng/mL). 24 h post stimulation, ECAR profiles, representing glycolysis, were determined utilising the Seahorse Extracellular Flux Analyser. (**L**) Summary of how the antimicrobials affect glycolytic profiles in unstimulated, iH37Rv-Mtb-stimulated and LPS-stimulated hMDMs. Bars denote mean ± SEM * *p* < 0.05 and ** *p* < 0.01 (Two-way repeated measures ANOVA with Sidak’s multiple comparisons test). Ethambutol (EMB, *n* = 7); isoniazid (INH, *n* = 8); pyrazinamide (PYZ, *n* = 7); rifampicin (RIF, *n* = 9); amikacin (AMK, *n* = 5); bedaquiline (BDQ, *n* = 6); clofazimine (CLO, *n* = 6); cycloserine (CYS, *n* = 4); linezolid (LIN, *n* = 8); moxifloxacin (MOX, *n* = 10).

**Figure 2:**
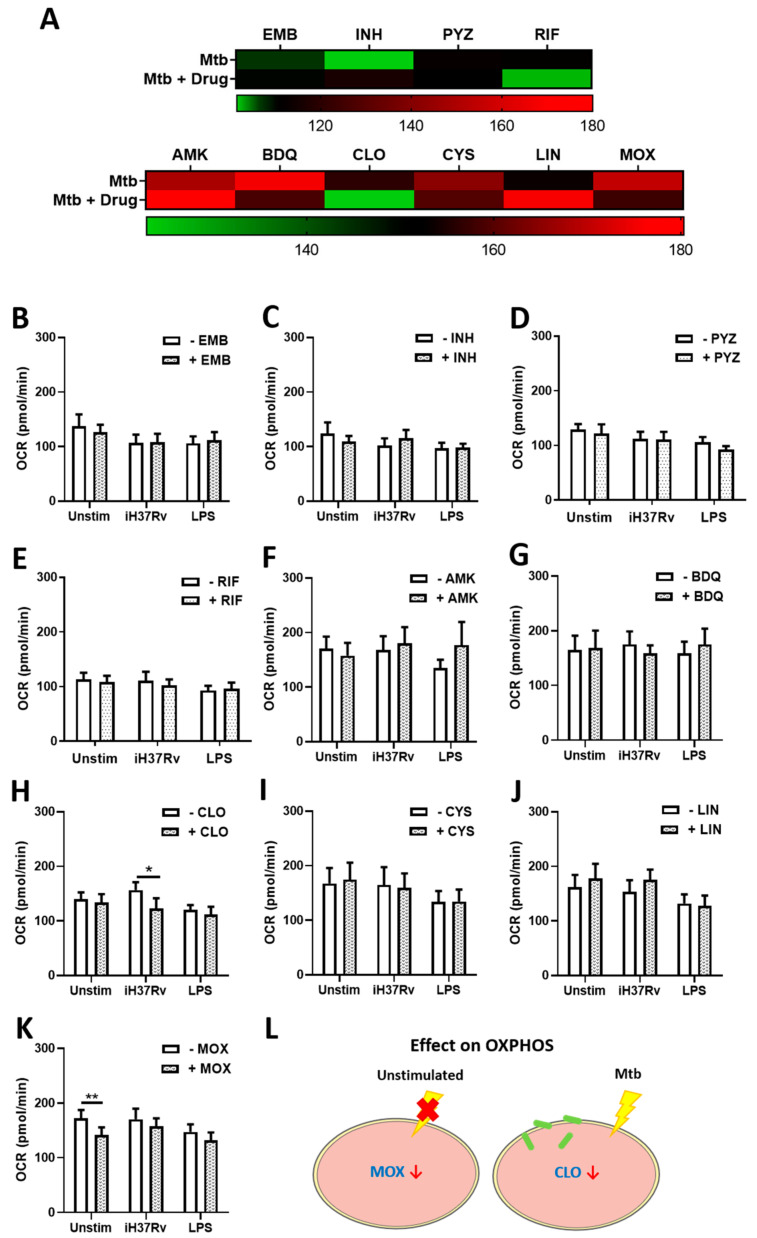
Moxifloxacin and clofazimine reduces oxidative phosphorylation in unstimulated and iH37Rv-Mtb-stimulated hMDMs, respectively. (**A**) Illustrative heap map showing alterations in OCR in response to treatment with first-line (top) and second-line (bottom) TB antimicrobials. hMDMs, differentiated from PBMCs isolated from healthy blood donors, were treated with (**B**) ethambutol (5 µg/mL), (**C**) isoniazid (1 µg/mL), (**D**) pyrazinamide (2 µg/mL), (**E**) rifampicin (2 µg/mL), (**F**) amikacin (5 µg/mL), (**G**) bedaquiline (5 µg/mL), (**H**) clofazimine (2 µg/mL), (**I**) cycloserine (5 µg/mL), (**J**) linezolid (15 µg/mL) or (**K**) moxifloxacin (12.5 µg/mL) three hours post stimulation with either iH37Rv-Mtb or LPS (100 ng/mL). 24 h post stimulation, OCR profiles, representing oxidative phosphorylation, were determined utilising the Seahorse Extracellular Flux Analyser. (**L**) Summary of how the antimicrobials affect glycolytic profiles in unstimulated, and iH37Rv-Mtb-stimulated hMDMs. Bars denote mean ± SEM * *p* < 0.05 and ** *p* < 0.01 (Two-way repeated measures ANOVA with Sidak’s multiple comparisons test). Ethambutol (EMB, *n* = 7); isoniazid (INH, *n* = 8); pyrazinamide (PYZ, *n* = 7); rifampicin (RIF, *n* = 9); amikacin (AMK, *n* = 5); bedaquiline (BDQ, *n* = 6); clofazimine (CLO, *n* = 6); cycloserine (CYS, *n* = 4); linezolid (LIN, *n* = 8); moxifloxacin (MOX, *n* = 10).

hMDMs, differentiated from peripheral blood mononuclear cells (PBMCs) isolated from healthy blood donors, were treated with one of four first-line (ethambutol (EMB), isonicotinic hydrazide; “isoniazid” (INH), pyrazinamide (PYZ) or rifampicin (RIF)) or six second-line (amikacin (AMK), bedaquiline (BDQ), clofazimine (CLO), cycloserine (CYS), linezolid (LIN), or moxifloxacin (MOX)) TB antimicrobials three hours post stimulation with either iH37Rv-Mtb or LPS. Use of the iH37Rv-Mtb strain was essential to negate any bactericidal effect of the TB antimicrobials on live Mtb. Concentrations of these TB antimicrobials, well documented in killing Mtb, were used as previously described [5,22,23,24,25,26,27]. At 24 h post stimulation, levels of glycolysis and OXPHOS were determined, measured by assessing extracellular acidification rate (ECAR) and oxygen consumption rate (OCR) respectively, utilising the Seahorse Extracellular Flux Analyser (Figure 1). We found that that TB antimicrobials differentially affected ECAR levels in hMDMs stimulated with iH37Rv-Mtb, illustrated by a heatmap showing iH37Rv-Mtb-stimulated hMDMs treated with and without TB antimicrobials (Figure 1A). Accordingly, the effect of the drugs is summarized on the heatmaps by comparing untreated Mtb-stimulated hMDMs on the top versus antimicrobial-treated Mtb-stimulated hMDMs on the bottom (green gradients represent low, black gradients represent intermediate and red gradients represent high levels of that output respectively). More specifically, we found that INH (Figure 1C), PYZ (Figure 1D), CLO (Figure 1H), and LIN (Figure 1J) significantly increased ECAR levels in iH37Rv-Mtb-stimulated hMDMs. EMB (Figure 1B), RIF (Figure 1E), AMK (Figure 1F), BDQ (Figure 1G), CYS (Figure 1I), and MOX (Figure 1K) did not affect ECAR levels. BDQ and CLO were the only antimicrobials to significantly increase ECAR in unstimulated hMDMs, whereas CLO was the only antimicrobial to increase ECAR in cells stimulated with LPS. CLO was the only antimicrobial to increase ECAR levels across all treatment conditions (Figure 1L). We also investigated the effect of the antimicrobials on OXPHOS by measuring OCR levels. Figure 2A summarises changes in OCR levels in iH37Rv-Mtb-stimulated hMDMs treated with (bottom) and without (top) the antimicrobials. We found that treatment with EMB (Figure 2B), INH (Figure 2C), PYZ (Figure 2D), RIF (Figure 2E), AMK (Figure 2F), BDQ (Figure 2G), CYS (Figure 2I), LIN (Figure 2J), and MOX (Figure 2K) did not alter OCR levels in hMDMs stimulated with iH37Rv-Mtb, however, CLO (Figure 2H) significantly reduced OCR levels in these cells. MOX was the only antimicrobial to significantly reduce OCR in unstimulated hMDMs. Antimicrobial treatment did not affect OCR levels in hMDMs stimulated with LPS. The effect of the antimicrobials on OCR is graphically summarised in Figure 2L.

**Figure 3:**
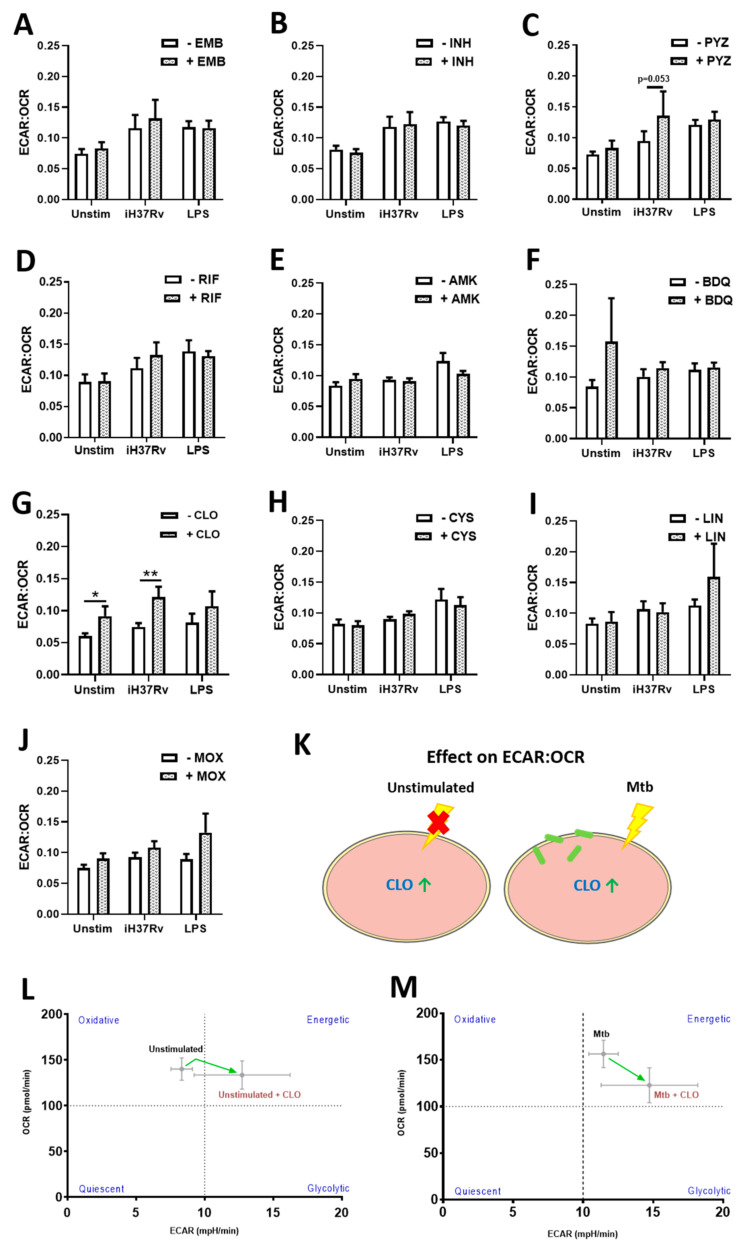
Clofazimine treated hMDMs stimulated with iH37Rv-Mtb rely on glycolysis by promoting Warburg metabolism. hMDMs, differentiated from PBMCs isolated from healthy blood donors, were treated with (**A**) ethambutol (5 µg/mL), (**B**) isoniazid (1 µg/mL), (**C**) pyrazinamide (2 µg/mL), (**D**) rifampicin (2 µg/mL), (**E**) amikacin (5 µg/mL), (**F**) bedaquiline (5 µg/mL), (**G**) clofazimine (2 µg/mL), (**H**) cycloserine (5 µg/mL), (**I**) linezolid (15 µg/mL) or (**J**) moxifloxacin (12.5 µg/mL) three hours post stimulation with either iH37Rv-Mtb or LPS (100 ng/mL). At 24 h post stimulation, the ECAR:OCR ratio was generated to measure the reliance of one metabolic pathway over another. (**K**) Summary of how clofazimine affects the ECAR:OCR ratio in unstimulated and iH37Rv-Mtb-stimulated hMDMs. This immunometabolic shift as a result of clofazimine treatment is illustrated by a metabolic phenogram in (**L**) unstimulated and (**M**) Mtb-stimulated hMDMs. Bars denote mean ± SEM * *p* < 0.05 and ** *p* < 0.01 (Two-way repeated measures ANOVA with Sidak’s multiple comparisons test). Ethambutol (EMB, *n* = 7); isoniazid (INH, *n* = 8); pyrazinamide (PYZ, *n* = 7); rifampicin (RIF, *n* = 9); amikacin (AMK, *n* = 5); bedaquiline (BDQ, *n* = 6); clofazimine (CLO, *n* = 6); cycloserine (CYS, *n* = 4); linezolid (LIN, *n* = 8); moxifloxacin (MOX, *n* = 10).

Next we determined the ratio of ECAR to OCR (ECAR:OCR), which allows us to determine a cell’s preference for glycolysis over OXPHOS (Figure 3). We found that treatment with EMB (Figure 3A), INH (Figure 3B), PYZ (Figure 3C), RIF (Figure 3D), AMK (Figure 3E), BDQ (Figure 3F), CYS (Figure 3H), LIN (Figure 3I), and MOX (Figure 3J) did not significantly affect this ratio in unstimulated hMDMs, or in hMDMs stimulated with iH37Rv-Mtb or LPS, meaning that these antimicrobial-treated hMDMs did not have a preference for either glycolysis or OXPHOS. We did find however, that CLO (Figure 3G) exhibited a significantly higher ratio indicative of a preference for glycolysis in unstimulated and iH37Rv-Mtb stimulated hMDMs (Figure 3K). This metabolic reliance and associated shift from oxidative metabolism to glycolytic metabolism in unstimulated and iH37Rv-Mtb stimulated hMDMs upon CLO treatment, is depicted by the phenograms in Figure 3L,M respectively.

### 2.2. Clofazimine Alters Mitochondrial Coupling Efficacy and Protein Leak in Primary hMDMs Stimulated with Mtb

**Figure 4:**
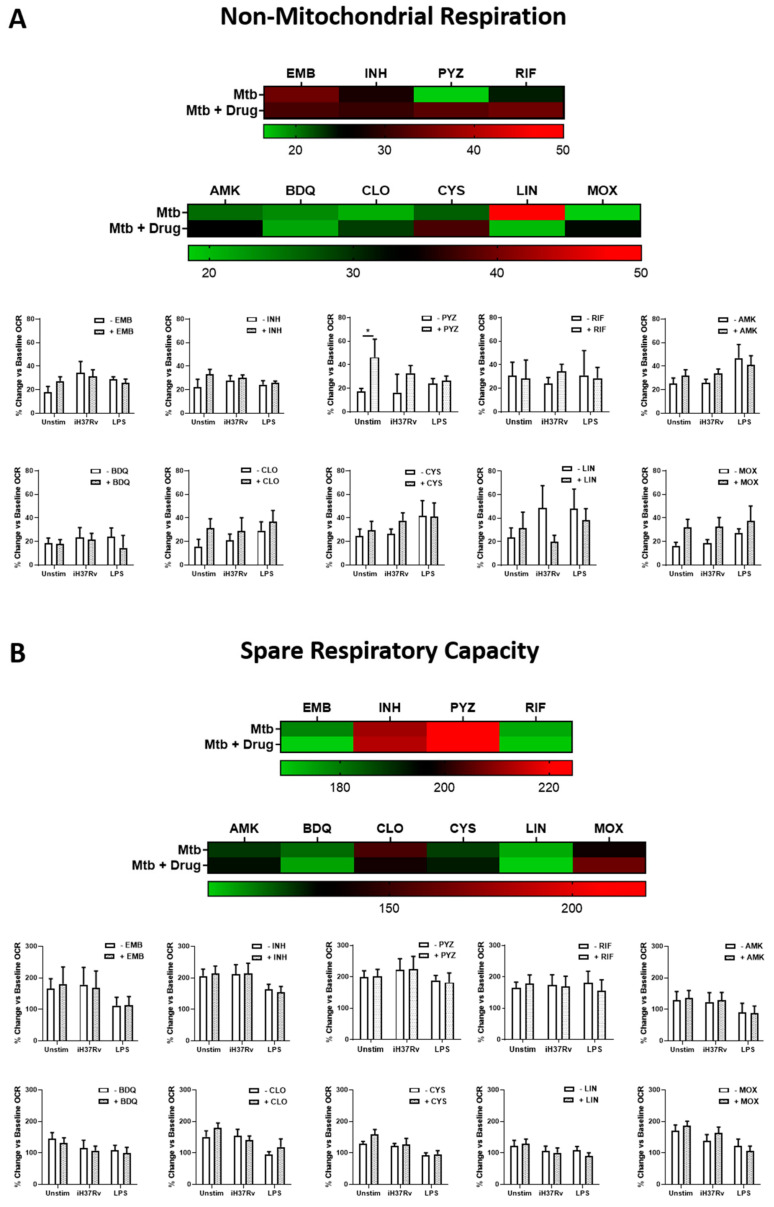
TB antimicrobials do not affect spare respiratory capacity or non-mitochondrial respiration in iH37Rv-Mtb-stimulated and LPS-stimulated hMDMs. hMDMs, differentiated from PBMCs isolated from healthy blood donors, were treated with ethambutol (5 µg/mL), isoniazid (1 µg/mL), pyrazinamide (2 µg/mL), rifampicin (2 µg/mL), amikacin (5 µg/mL), bedaquiline (5 µg/mL), clofazimine (2 µg/mL), cycloserine (5 µg/mL), linezolid (15 µg/mL) or moxifloxacin (12.5 µg/mL) three hours post stimulation with either iH37Rv-Mtb or LPS (100 ng/mL). 24 h post stimulation, (**A**) non-mitochondrial respiration and (**B**) spare respiratory capacity were determined utilising the Seahorse Extracellular Flux Analyser. Bars denote mean ± SEM * *p* < 0.05 (Two-way repeated measures ANOVA with Sidak’s multiple comparisons test). Ethambutol (EMB, *n* = 7); isoniazid (INH, *n* = 6); pyrazinamide (PYZ, *n* = 7); rifampicin (RIF, *n* = 9); amikacin (AMK, *n* = 5); bedaquiline (BDQ, *n* = 6); clofazimine (CLO, *n* = 6); cycloserine (CYS, *n* = 4); linezolid (LIN, *n* = 8); moxifloxacin (MOX, *n* = 10).

**Figure 5:**
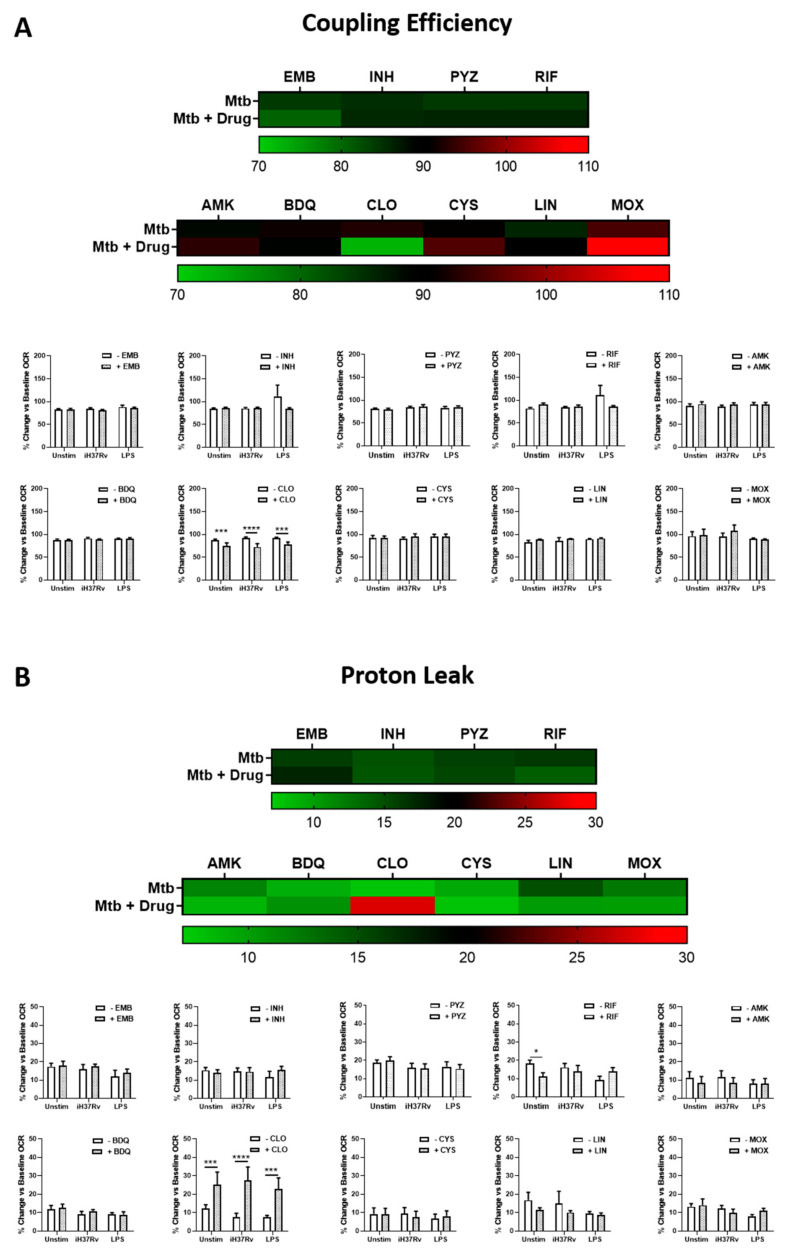
Clofazimine reduces mitochondrial coupling efficiency and increases mitochondrial proton leak in iH37Rv-Mtb-stimulated hMDMs. hMDMs, differentiated from PBMCs isolated from healthy blood donors, were treated with ethambutol (5 µg/mL), isoniazid (1 µg/mL), pyrazinamide (2 µg/mL), rifampicin (2 µg/mL), amikacin (5 µg/mL), bedaquiline (5 µg/mL), clofazimine (2 µg/mL), cycloserine (5 µg/mL), linezolid (15 µg/mL) or moxifloxacin (12.5 µg/mL) three hours post stimulation with either iH37Rv-Mtb or LPS (100 ng/mL). 24 h post stimulation, (**A**) mitochondrial coupling efficiency and (**B**) mitochondrial proton leak were determined using the Seahorse Extracellular Flux Analyser. Bars denote mean ± SEM * *p* < 0.05 (Two-way repeated measures ANOVA with Sidak’s multiple comparisons test). Ethambutol (EMB, *n* = 7); isoniazid (INH, *n* = 8); pyrazinamide (PYZ, *n* = 7); rifampicin (RIF, *n* = 9); amikacin (AMK, *n* = 5); bedaquiline (BDQ, *n* = 6); clofazimine (CLO, *n* = 6); cycloserine (CYS, *n* = 4); linezolid (LIN, *n* = 8); moxifloxacin (MOX, *n* = 10).

As metabolism is intrinsically linked with mitochondrial function, it was also necessary to examine how these antimicrobials affected those facets of mitochondrial function which are more closely linked to energy production. hMDMs were treated with the TB antimicrobials three hours post stimulation with either iH37Rv-Mtb or LPS as before. At 24 h post stimulation, non-mitochondrial respiration, spare respiratory capacity, maximum respiratory capacity, coupling efficiency (or ATP production), and protein leak were determined utilising the Seahorse Extracellular Flux Analyser as previously described [29]. First, we ensured any antimicrobial-induced alterations in oxygen consumption, as measured by OCR, were not driven by processes outside the mitochondria. Even though PYZ significantly increased non-mitochondrial respiration in unstimulated hMDMs, we found that non-mitochondrial respiration was not significantly different upon antimicrobial treatment in unstimulated, iH37Rv-Mtb stimulated, and LPS-stimulated hMDMs (Figure 4A). This indicates that the TB antimicrobials do not promote any other processes of oxygen consumption outside the mitochondria, and that any antimicrobial-induced alterations in metabolism can be attributed to the mitochondria themselves. We also examined if antimicrobial treatment could promote augmented respiration, by assessing spare respiratory capacity in the same cells. We found that none of the antimicrobials increased spare respiratory capacity in unstimulated, iH37Rv-Mtb stimulated, and LPS-stimulated hMDMs (Figure 4B). Next, we assessed if any of the TB antimicrobials directly affected the ability of the cells to produce ATP, as measured through their coupling efficiency. We found that CLO significantly reduced coupling efficiency in unstimulated, iH37Rv-Mtb stimulated, and LPS-stimulated hMDMs (Figure 5A), supporting our earlier finding that CLO treatment promotes a metabolic shift from OXPHOS to glycolysis. Coupling efficiency was unaffected upon treatment with the remaining nine antimicrobials. To interrogate this further, we examined if these TB antimicrobials could increase proton leak in these hMDMs, as a common cause of mitochondrial uncoupling can be increased proton leak. The process of making ATP through OXPHOS involves pumping protons across the mitochondrial membrane; the subsequent proton gradient that this pumping forms drives the synthesis of ATP. Any increases in proton leak can dissipate this proton gradient and significantly hamper mitochondrial coupling efficiency. We found that CLO treatment significantly increased proton leak in unstimulated, iH37Rv-Mtb stimulated, and LPS-stimulated hMDMs (Figure 5B), with reduced proton leak observed in unstimulated hMDMs treated with PYZ.

In summary, while CLO-induced increases in proton leak still contribute to oxygen being consumed, CLO treatment leads to increased proton leak and reduced mitochondrial uncoupling (i.e., reduced ATP production) in unstimulated, iH37Rv-Mtb-stimulated, and LPS-stimulated hMDMs TB antimicrobials did not promote non-mitochondrial respiration and did not affect spare respiratory capacity in unstimulated, iH37Rv-Mtb-stimulated, or LPS-stimulated hMDMs.

### 2.3. TB Antimicrobials Differentially Affect Levels of Secreted Cytokines and Chemokines in Unstimulated, iH37Rv-Mtb Stimulated and LPS-Stimulated hMDMs

**Figure 6:**
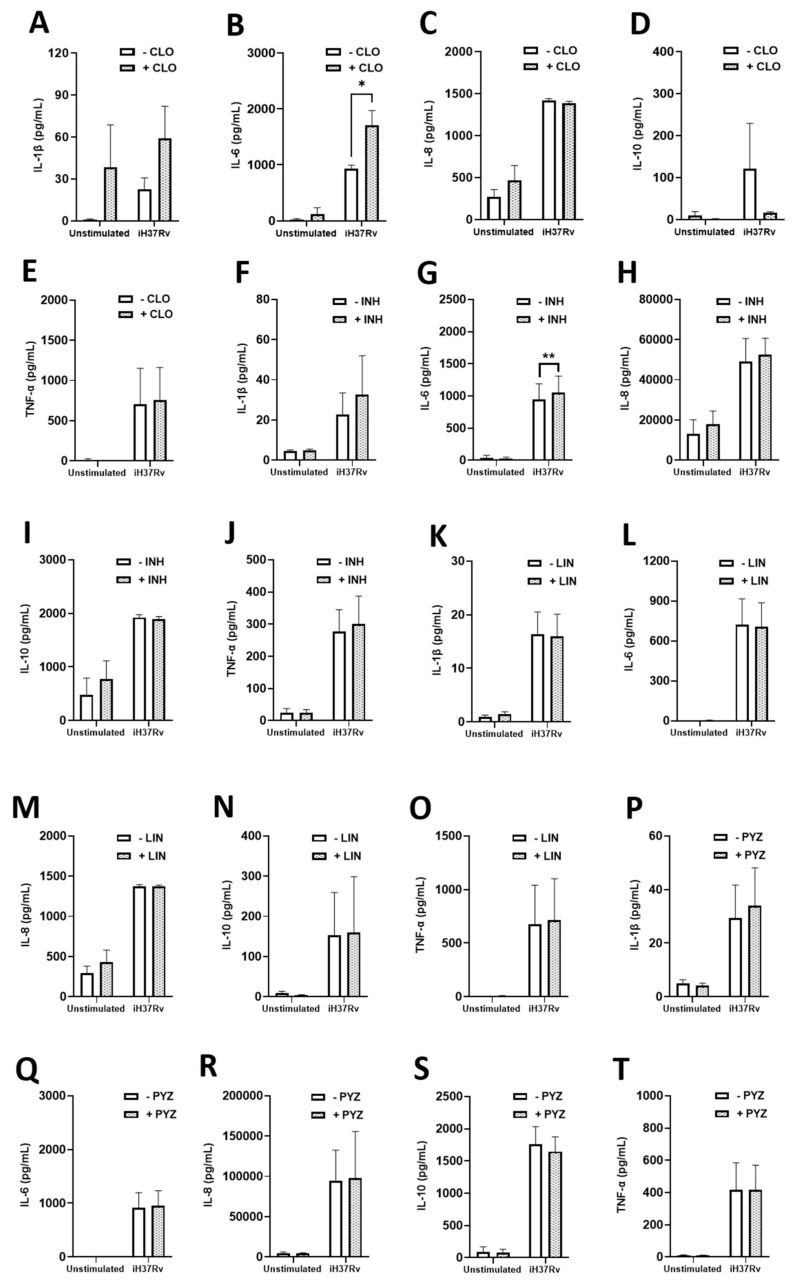
Examining the effect of clofazimine, isoniazid, linezolid and pyrazinamide on protein levels of IL-1β, IL-6, IL-8, IL-10 and TNFα in hMDMs stimulated with iH37Rv-Mtb. hMDMs, differentiated from PBMCs isolated from healthy blood donors, were stimulated with iH37Rv *Mtb* for three hours, washed to remove unphagocytosed *Mtb,* and were treated with clofazimine (2 µg/mL), isoniazid (1 µg/mL), linezolid (15 µg/mL) or pyrazinamide (2 µg/mL). At 24 h post stimulation, protein levels of IL1β (**A**,**F**,**K** and **P**), IL-6 (**B**,**G**,**L** and **Q**), IL-8 (**C**,**H**,**M** and **R**), IL-10 (**D**,**I**,**N** and **S**) and TNFα (**E**,**J**,**O** and **T**) were quantified using Meso Scale Discovery Multi-Array technology. Bars denote mean ± SEM. * *p* < 0.05 (Two-way repeated measures ANOVA tests with Šídák’s multiple comparisons tests).

**Figure 7:**
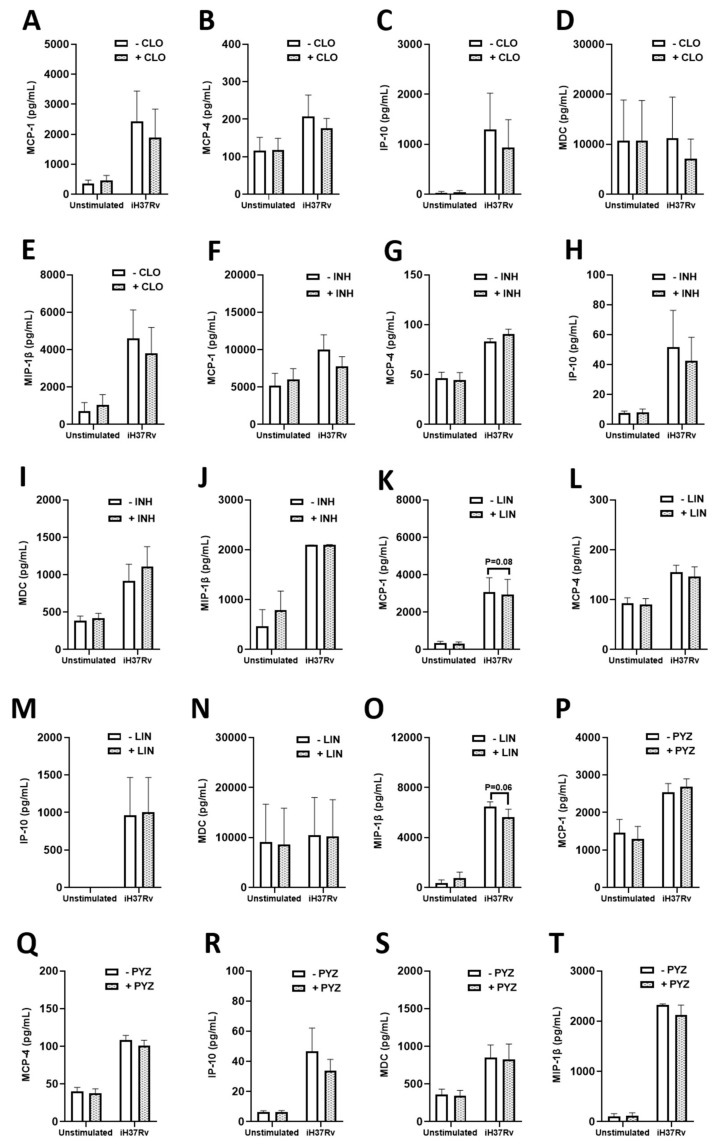
Assessing the effect of clofazimine, isoniazid, linezolid and pyrazinamide on protein levels of MCP-1, MCP-4, IP-10, MDC and MIP-1β in hMDMs stimulated with iH37Rv-Mtb. hMDMs, differentiated from PBMCs isolated from healthy blood donors, were stimulated with iH37Rv-Mtb for three hours, washed to remove unphagocytosed Mtb, and were treated with clofazimine (2 µg/mL), isoniazid (1 µg/mL), linezolid (15 µg/mL) or pyrazinamide (2 µg/mL). At 24 h post stimulation, protein levels of MCP-1 (**A**,**F**,**K** and **P**), MCP-4 (**B**,**G**,**L** and **Q**), IP-10 (**C**,**H**,**M** and **R**), MDC (**D**,**I**,**N** and **S**) and MIP-1β (**E**,**J**,**O** and **T**) were quantified using Meso Scale Discovery Multi-Array technology. Bars denote mean ± SEM. * *p* < 0.05 (Two-way repeated measures ANOVA tests with Šídák’s multiple comparisons tests).

As immunometabolism, particularly glycolysis, underpins the immune response to Mtb infection and can mediate the secretion of a variety of cytokines and chemokines in hMDMs, we wanted to further examine if some of these antimicrobials could be regulating the production of any secreted factors in our model. To do this, we quantified the levels of various chemokines and cytokines in the matched supernatants from the metabolic analyses, specifically by those antimicrobials we found to significantly increase glycolysis, namely INH, PYZ, CLO, and LIN. Some of the chemokines and cytokines assayed have also been shown to indirectly and directly kill Mtb, while some help to recruit additional immune cells to the site of infection in vivo thereby promoting mycobacterial clearance. We treated hMDMs with INH, PYZ, CLO, and LIN three hours post stimulation with either iH37Rv-Mtb or LPS. Twenty-four hours post stimulation, cell supernatants were collected and levels of the cytokines IL-1β, IL-6, IL-8, IL-10, and TNFα were assessed (Figure 6). We found that CLO (Figure 6B) and INH (Figure 6G) significantly increased IL-6 levels in hMDMs stimulated with iH37Rv-Mtb. LIN (Figure 6L) and PYZ (Figure 6Q) did not affect IL-6 levels in the same cells. Levels of IL-1β (Figure 6A,F,K,P), IL-8 (Figure 6C,H,M,R), IL-10 (Figure 6D,I,N,S), and TNFα (Figure 6E,J,O,T) were not significantly affected by CLO, INH, LIN, and PYZ treatment in iH37Rv-Mtb-stimulated hMDMs, respectively. Moreover, we found that CLO, INH, LIN, and PYZ did not significantly affect secreted levels of IFN-γ (Supplementary Figure S1A,F,K,P), IL-12 (Supplementary Figure S1B,G,L,Q), IL-13 (Supplementary Figure S1C,H,M,R), IL-2 (Supplementary Figure S1D,I,N,S), and IL-4 (Supplementary Figure S1E,J,O,T), respectively. Next, we assessed how these antimicrobials affected chemokine levels. We found that CLO, INH, LIN, and PYZ did not alter MCP-1 (Figure 7A,F,K,P), MCP-4 (Figure 7B,G,L,Q), IP-10 (Figure 7C,H,M,R), MDC (Figure 7D,I,N,S), and MIP-1β (Figure 7E,J,O,T) levels in iH37Rv-Mtb stimulated hMDMs, respectively. Furthermore, CLO, INH, LIN, and PYZ did not affect secreted levels of Eotaxin (Supplementary Figure S2A,E,H,L), Eotaxin-3 (Supplementary Figure S2B,F,I,M), MIP-1α (Supplementary Figure S2C,J), or TARC (Supplementary Figure S2D,G,K,N).

It is now well established that Mtb strains are recognized by a variety of toll-like receptors resulting in differential immune responses (5). Moreover, some evidence suggests that distinct TLR recognition evoked by different Mtb strains may be decisive for shaping innate immune responses in vitro and in vivo, and possibly contribute to the outcome of Mtb infection (5). Therefore, establishing how these antimicrobials also affect hMDMs stimulated with the toll-like receptor-4 agonist LPS, could help to interpret different mechanisms of action in our models. Using matched supernatants from the metabolic analyses, we found that unstimulated hMDMs and LPS-stimulated hMDMs, treated with CLO exhibited significantly higher levels of IL-1β (Supplementary Figure S3A). INH (Supplementary Figure S3F), LIN (Supplementary Figure S3K), and PYZ (Supplementary Figure S3P) did not alter IL-1β levels in these LPS-stimulated hMDMs. Levels of IL-6 (Supplementary Figure S3B,G,L,Q), IL-8 (Supplementary Figure S3C,H,M,R), IL-10 (Supplementary Figure S3D,I,N,S), and TNFα (Supplementary Figure S3E,J,O,T) were not significantly affected by CLO, INH, LIN, and PYZ treatment in LPS-stimulated hMDMs, respectively. Moreover, we found that CLO, INH, LIN, and PYZ did not significantly affect secreted levels of IFN-γ (Supplementary Figure S4A,F,K,P), IL-12 (Supplementary Figure S4B,G,L,Q), IL-13 (Supplementary Figure S4C,H,M,R), IL-2 (Supplementary Figure S4D,I,N,S), and IL-4 (Supplementary Figure S4E,J,O,T), respectively. We did find, however, that the antimicrobials exhibited profound effects on chemokine secretion in hMDMs stimulated LPS. CLO significantly reduced MCP-4 (Supplementary Figure S5B), MDC (Supplementary Figure S5D), and TARC (Supplementary Figure S6D) levels without affecting MCP-1, IP-10, MIP-1β, Eotaxin, Eotaxin-3, and MIP-1α (Supplementary Figures S5A,C,E and S6A–C, respectively). INH increased MCP-1 levels (Supplementary Figure S5F) without affecting the levels of the other chemokines (Supplementary Figures S5G–J and S6E–G). Interestingly, LIN significantly reduced levels of MCP-1, MCP-4, IP-10, MIP-1β, and MIP-1 (Supplementary Figures S5K,L,M,O and 6J, respectively) without affecting levels of MDC, Eotaxin, Eotaxin-3, and TARC (Supplementary Figures S5N and S6H,I,K, respectively). PYZ did not alter chemokine levels in hMDMs stimulated with LPS (Supplementary Figures S5P–T and S6L–N).

In summary, CLO increases ECAR in unstimulated, iH37Rv-Mtb-stimulated, and LPS-stimulated hMDMs and decreases OCR in iH37Rv-Mtb-stimulated hMDMs but not in LPS-stimulated hMDMs. CLO treatment of hMDMs also induces an increase in IL-6 secretion during iH37Rv-Mtb stimulation but not when stimulated with LPS, suggesting a link between IL-6 secretion and Warburg metabolism in CLO-treated hMDMs. Interestingly, CLO treatment increased IL-1β secretion levels in LPS-stimulated hMDMs.

## 3. Discussion

For over fifty years now, TB patients have undergone long, complex drug treatment regimens involving the use of several antimicrobials. It is widely understood that these antimicrobials have direct bactericidal effects, however very little is known about their role in modulating the functions of host immune cells, including macrophages which are the earliest recruited immune cell to the site of infection. The continued rise in Mtb resistance against first- and second-line antimicrobials has led to increasing research around host directed therapies, which aim to improve TB treatment by enhancing host immune responses [4,6,30]. A limited number of studies have successfully detailed how some TB antimicrobials mediate host immune responses to TB, such as altering autophagic flux and the secretion of various cytokines and chemokines [21,31,32,33,34,35]. In our current study, we show for the first time that first- and second-line TB antimicrobials can differentially modulate the host immune response, altering glycolysis, OXPHOS, mitochondrial function (including mitochondrial coupling efficiency and proton leak), and cytokine and chemokine secretions in primary hMDMs stimulated with iH37Rv-Mtb and LPS. We show how CLO induces a metabolic shift similar to the Warburg effect, increasing glycolysis and decreasing OXPHOS in iH37Rv-Mtb-stimulated hMDMs. CLO treatment of hMDMs also induced an increase in IL-6 secretion during iH37Rv-Mtb stimulation but not when stimulated with LPS. These interesting findings suggest a link between IL-6 and a shift towards glycolytic metabolism in CLO-treated macrophages during TB infection.

Immunometabolism underpins the host’s immune response to Mtb infection, and it is well known that enhanced glycolytic flux activity facilitates early Mtb clearance by the innate immune system, as well as facilitating the production of crucial proinflammatory cytokines [5,10,36]. More pertinently, the augmentation of glycolysis has been identified as one important strategy in the development of host-directed therapies for TB [4,6]. Macrophages are the first immune cells to encounter Mtb during infection, thus their function is crucial for the early clearance of Mtb. In the current study, we show that INH, PYZ, LIN, and CLO significantly increased glycolysis in hMDMs stimulated with iH37Rv-Mtb. Some of these antimicrobials have been shown to alter glycolytic flux in other cell models. LIN treatment, which is often associated with lactic acidosis, increases glycolytic activity in primary human osteoblasts, measured by an increase in lactate production [37]. This effect can be attributed to the inhibition of mitochondrial protein synthesis which reduces mitochondrial respiration and increases anaerobic glycolysis leading to the overproduction of lactate [20,37]. However, as decreased OXPHOS levels by LIN were not detected in our model, this increase in glycolytic activity is likely due to factors other than a result of mitochondrial dysfunction. INH is also associated with impairments in mitochondrial function, as INH treatment reduces the activity of mitochondrial complexes in rat brain and liver mitochondria leading to reduced ATP production [13]. However, again, we did not observe similar mitochondrial impairments or reduced OXPHOS activity in our Mtb model, indicating that an increase in glycolytic activity alone is attainable with INH treatment. PYZ treatment is associated with increased reactive oxygen species (ROS) production in zebrafish larvae [15], however it is unknown if mitochondrial ROS contributes to this. Again, our cell model did not indicate changes in OXPHOS or mitochondrial function upon treatment of hMDMs with PYZ, further suggesting that increased glycolytic activity can be stimulated with the use of PYZ. CLO has been previously shown to be associated with a shift towards glycolytic metabolism in a murine model, accompanied by decreased urinary excretion of TCA cycle intermediates and increased lactate:glucose ratios [38]. CLO treatment also significantly increased glycolytic activity in unstimulated and LPS-stimulated hMDMs, indicating that CLO-induced shifts to glycolysis in hMDMs may be augmented through TLR4 [39]. However, it is likely that other signalling pathways are involved in increasing glycolytic flux in these cells, as evidenced by similar ECAR levels in unstimulated and LPS-stimulated hMDMs upon CLO treatment, and the effect of CLO on mitochondrial uncoupling and proton leak across all treatment conditions. As INH, PYZ, LIN, and CLO can augment glycolysis in hMDMs, use of these specific antimicrobials could also aid in promoting early clearance of Mtb, while avoiding mitochondrial dysfunction and any associated side effects exhibited by more toxic antimicrobials.

We next examined if these TB antimicrobials could alter mitochondrial OXPHOS. We found that CLO significantly decreased OXPHOS in hMDMs stimulated with iH37Rv-Mtb. In human non-small-cell bronchial-carcinoma cells, when treated with CLO, ATP production switches from OXPHOS to a more glycolytic-dependent means of ATP production [19]. This is associated with mitochondrial dysfunction and increased lactate levels, indicative, again, of increased glycolytic activity [19]. This corresponds with our current findings. In fact, CLO also exhibits a significant preference for glycolysis over OXPHOS in unstimulated and iH37Rv-Mtb-stimulated hMDMs. This immunometabolic shift from OXPHOS to glycolysis is reminiscent of the Warburg effect and is commonly associated with the rapid generation of ATP and biosynthetic precursors. Inducing this bioenergetic shift, through the use of CLO, could potentially be harnessed for adequate early clearance of Mtb.

Next, we wanted to determine if these changes in OXPHOS and glycolysis were associated with alterations in mitochondrial function, as optimal mitochondrial function contributes to multiple biological functions, including the production of ATP [40]. We found that CLO treatment reduced mitochondrial coupling efficiency (ATP production) and concomitantly increased proton leak in unstimulated, iH37Rv-Mtb-stimulated, and LPS-stimulated hMDMs. Such effects on mitochondrial coupling efficiency and proton leak would likely explain reduced ATP production, and a preference for glycolytic metabolism in CLO-treated hMDMs. ATP production through the process of OXPHOS involves pumping protons across the mitochondrial membrane; the subsequent proton gradient that this pumping forms drives the synthesis of ATP, thus any leak would reduce the ability of hMDMs to produce ATP. Indeed, CLO is known for its mitochondrial uncoupling activity in human brain cells and energy metabolism in human lung cancer cells and it is also believed that overcoming the development of drug resistance is more feasible using drugs that specifically target the physical properties of membranes, such as the mitochondrial membrane potential and its ability to couple efficiently [41]. This further supports the observed CLO-induced decrease in OXPHOS and may explain the increased requirement to redirect cellular processes and pathways towards glycolysis-dependent ATP production.

BDQ and INH have previously been reported to suppress mitochondrial function in rat models, specifically by inhibiting the mitochondrial electron transport chain [13,17]. EMB is also known to be toxic to mitochondria by inducing a mitochondrial coupling defect and inhibiting complex IV [12]. Co-treatment of INH and RIF induces oxidative stress in the mitochondria and increased mitochondrial permeability transition in mice [14]. Interestingly, in epithelial cells, RIF is thought to mainly target the mitochondria, whereby it induces pathological changes to the structure of the mitochondria resulting in excessive production of ROS and the release of cytochrome c [16]. However, in our model using primary hMDMs stimulated with Mtb, we did not observe any statistically significant changes in mitochondrial function as marked by the OCR, spare respiratory capacity, coupling efficiency or proton leak induced by BDQ, INH, EMB, or RIF.

CLO has been shown to accumulate in the mitochondria of kidney cells and can alter metabolic function in lung fibroblast cells [19,42]. This inhibition of mitochondrial function by CLO was also associated with increased lactate production, indicative of a compensatory increase in glycolysis in the cancer lung fibroblast cell line [19]. This is in keeping with our findings in primary human macrophages showing that CLO significantly increased flux through glycolysis in both resting and stimulated macrophages, whereas as a statistically significant decrease on OCR was only observed in the context of stimulation with Mtb.

As glycolysis underpins the immune response to Mtb infection and can mediate the secretion of various cytokines and chemokines in hMDMs, we wanted to further examine if some of these antimicrobials could also be regulating the production of secreted factors in our model. To do this, we examined protein secretions from those antimicrobials we found to significantly alter glycolysis the most, that is INH, PYZ, LIN, and CLO. For the most part, these antimicrobials did not alter cytokine or chemokine levels in iH37Rv-Mtb-stimulated hMDMs. iH37Rv-Mtb stimulated hMDMs treated with INH and CLO induced higher levels of IL-6. IL-6 is a crucial cytokine during Mtb infection and early studies show that IL-6 deficient mice develop deadly TB symptoms [43]. IL-6 has been shown to promote Th1, Th2, cytotoxic T cell, and NK cell responses [44,45,46] and has recently been attributed to enhancing glycolysis in cancer cell models [47,48]. Therefore, although we aimed to determine cytokine alterations caused by changes in immunometabolism, IL-6 increases may not be a direct result but could instead be an attributed cause for the increase in glycolytic activity observed in response to INH and CLO treatment. In fact, in mouse embryonic fibroblasts and human cell lines, IL-6 activates STAT3, enhancing glycolytic enzymes and increasing glycolytic flux [48]. Interestingly, IL-6 was not increased in LPS-stimulated hMDMs treated with CLO or INH, also confirming that this is not a TLR4-mediated process. Furthermore, the absence of an increase in IL-6 secretion correlated to increased ECAR but no decrease in OCR in CLO-treated, LPS-stimulated hMDMs. However, increased IL-6 secretion was observed in CLO-treated, iH37Rv-Mtb-stimulated hMDMs and this was associated with both an increase in ECAR and a decrease in OCR. This novel finding suggests that IL-6 secretion is linked not only to an increase in glycolysis but to the Warburg effect in hMDMs.

Interestingly, there was no noticeable increase in IL-1β, TNF-α, or IL-8 in hMDMs treated with INH, PYZ, LIN, or CLO. Although these cytokines are important in the early proinflammatory response to Mtb, failure to induce these factors in patients with active TB on these antimicrobials is likely beneficial as inflammation-mediated tissue damage and other possible harmful side effects will be avoided. Increases in IL-1β were observed however, in LPS-stimulated hMDMs treated with CLO, suggesting that this is due to a more specific stimulus through TLR4, compared to the IL-6 response in hMDMs stimulated with iH37Rv-Mtb which likely involves multiple TLR stimulants and/or other pathways. Thus, since CLO treatment increases both glycolytic activity and IL-1 β secretion in LPS-stimulated hMDMs, our study suggests a use for CLO as a HDT in the treatment of gram-negative bacteria.

We also determined the effect of these antimicrobials on chemokine secretion in iH37Rv-Mtb- and LPS-stimulated hMDMs. We found that CLO elicits anti-chemotactic effects, decreasing the secretion of MCP-4, MDC, and TARC in LPS-stimulated hMDMs but not in iH37Rv-Mtb-stimulated hMDMs. MCP-4 is a chemoattractant for monocytes and T cells, and known to induce granuloma formation [49], MDC is an attractant for Th2 cells, monocytes, monocyte-derived dendritic cells and natural killer cells [50], and TARC is a selective attractant to Th2 cells [51]. Furthermore, LIN treatment significantly reduced protein levels of MCP-1, MCP-4, IP-10, and MIP-1β. Like CLO, LIN-induced chemokine alterations were not observed in iH37Rv-stimulated hMDMs in LPS-stimulated hMDMs. Since the decline in chemokine levels were not recapitulated in our iH37Rv-Mtb hMDM model, it is possible that potent activation of TLR4 is an important mediator in the chemotaxic process in the LPS-hMDM model, even though multiple TLRs are likely engaged in Mtb-stimulated hMDMs and during CLO and LIN treatment, but perhaps not to the same magnitude. It is also possible that engaging multiple TLRs simultaneously has both additive and preventative downstream effects in regulating the expression of these chemokines, which could also act to cancel each other out to some extent. As LPS stimulation also represents a sepsis model, these finding are also useful, whereby CLO and LIN exhibit anti-chemotactic effects, reducing immune cell recruitment and inflammation during sepsis, while still augmenting immunometabolic processes in the process.

Our data shows that current TB antimicrobials differentially alter bioenergetics, mitochondrial function and immune responses in iH37Rv-Mtb- and LPS-stimulated hMDMs, expanding on their primary bactericidal functions. These findings suggest that antimicrobial-induced effects could be beneficial in patients harbouring bacterial resistance as they can elicit host-directed functions, for example, through the CLO-induced reprogramming of immunometabolism which can enhance immune function and facilitate Mtb clearance [10]. Substantial knowledge gaps remain in the mechanistic and functional downstream effects of altered energetics in our system. Our work has previously shown that altered cellular energetics in human macrophages are closely associated with inflammatory function, autophagy, and bacillary clearance, for example [4,6,10,52,53]. As such, future studies should also seek to examine if these antimicrobials can recapitulate the immunomodulatory findings in the current study and enhance Mtb clearance in suitable primary human and in vivo mouse models harbouring live resistant Mtb strains. These and other studies should also examine if additional processes, such as autophagy, can be enhanced using some of these TB antimicrobials. While some studies suggest that immunometabolic reprogramming could have negative effects, resulting in the overproduction of various pro-inflammatory mediators and heightened immune cell exhaustion in the late stages of infection [54], our findings show that these drugs can support hMDM metabolism, without inducing an unwanted cytokine storm. The current data highlights emerging prospects in repurposing current TB antimicrobials, as their immunomodulatory effects on host function could still be therapeutically beneficial even in patients with drug-resistant TB [1]. Accordingly, due to their ability to directly augment immune function, some of these antimicrobials could also help infected hMDMs to fight a variety of both bacterial and viral infections. This highlights the possibility of improving the utilization of these long-established antimicrobials for patients with TB, which could also help to pave the way for more stratified treatment regimens in the future.

## 4. Materials and Methods

### 4.1. hMDM Cell Culture

Peripheral blood buffy coats were obtained from the Irish Blood Transfusion Services in Dublin, Ireland. PBMCs were isolated by density gradient centrifugation with Lymphoprep^TM^ (Stemcell Technologies, Vancouver, BC, Canada) and seeded at 2.5 × 10^6^ cells/mL in Roswell Park Memorial Institute (RPMI) 1640 medium (Bio-Sciences, Nottingham, UK), supplemented with 10% AB-human serum (Sigma-Aldrich, St. Louis, MO, USA) and plated onto non-treated cell culture plates (Corning). LabTeks^TM^ (Nunc, Roskilde, Denmark) were also seeded to determine the multiplicity of infection (MOI; see Section 4.2). To obtain hMDMs, the cells were cultured over 7 days at 37 °C and 5% CO_2_ to allow differentiation prior to experimentation. Cells were washed every 2–3 days to remove non-adherent cells. On day 7, hMDMs were routinely greater that 90% pure, as determined by flow cytometry-based analysis of CD14 and CD68 co-expression (data not shown).

### 4.2. Stimulation of hMDMs with iH37Rv-Mtb

On the day of infection, log phase iH37Rv-Mtb was centrifuged at 3000× *g* for 10 min and resuspended in RPMI 1640 medium (supplemented with 10% AB-human serum). The suspension was passed 10 times through a 25-gauge needle and centrifuged at 100× *g* for 3 min to remove any bacterial clumps. The volume of bacterial suspension required for a given MOI was determined by treating macrophages with a range of volumes of resuspended Mtb. hMDMs in Labteks^TM^ were incubated with iH37Rv-Mtb for 3 h, washed with pre-warmed PBS to remove extracellular bacteria and fixed with 2% paraformaldehyde (PFA) (Sigma-Aldrich) for 10 min. hMDMs were subsequently stained with Modified Auramine O stain and Modified Auramine O decolorizer (Scientific Device Laboratory, Des Plaines, IL, USA) followed by Hoechst 33242 (Sigma-Aldrich) to counterstain the nuclei. The cells were analysed under an inverted fluorescent microscope (Olympus IX51) to determine the average number of phagocytosed bacilli per cell and percentage of cells infected. The required volume of bacilli was determined, phagocytic variation was adjusted between donors to ensure the same MOI (1–10 bacilli/cell, 70% positivity approximately) and the calculated volume of resuspendediH37Rv- Mtb added to the appropriate experimental wells.

Next, 3 h later, extracellular bacteria were washed off with PBS, fresh complete RPMI was added and TB antimicrobials were added; 5 µg/mL ethambutol (in dH_2_O), 1 µg/mL isoniazid (in dH_2_O), 2 µg/mL pyrazinamide (in dH_2_O), 5 µg/mL cycloserine (in dH_2_O), 5 µg/mL bedaquiline (in DMSO) (Medchem Express, Monmouth Junction, NJ, USA), 2 µg/mL rifampicin (in dH_2_O) (Fisher Scientific, Waltham, MA, USA), 5 µg/mL amikacin (in dH_2_O), 2 µg/mL clofazimine (in DMSO), 15 µg/mL linezolid (in DMSO), or 12.5 µg/mL moxifloxacin (in dH_2_O; Sigma-Aldrich). Macrophages were incubated for a further 21 h (24 h total). All drugs were assayed in parallel with their own equivalent volumes of vehicle control. Non-treated and antimicrobial-treated unstimulated hMDMs were also assayed in parallel. Un-treated/stimulated, antimicrobial-treated, and LPS (100 ng/mL)-stimulated hMDMs were also assayed in parallel as controls, and also to gain insight into how these antimicrobials may affect these cellular processes in the context of other bacterial infections.

### 4.3. Estimating Cell Number and Cell Viability Using Crystal Violet and Propidium Iodide (PI) Based Cell Exclusion Assays

hMDMs were stimulated with iH37Rv-Mtb and treated with TB antimicrobials, as described above. Cell viability was determined using a PI based cell exclusion assay. Cells were stained with 5 µg/mL PI (Sigma-Aldrich), 20 µg/mL Hoechst 33342 (Sigma-Aldrich) and 50 µg/mL Hoechst 33258 (Sigma-Aldrich) for 30 min at room temperature. Total cell numbers were detected via Hoechst staining of nuclei (Blue channel: Ex 390 nm/Em 430 nm) and dying/dead cells were identified via positivity for PI staining (Orange channel: Ex 544 nm/EM 588 nm), using the Lionheart™ FX Automated Microscope Imaging System (Bio-Tek, Winooski, VT, USA). Five fields of view were acquired per treatment per well.

Relative cell numbers were determined using a Crystal Violet assay. hMDMs were fixed with 1% glutaraldehyde (in PBS) for 15 min at room temperature and washed with PBS. hMDMs were then incubated at room temperature for 20 min with 0.1% crystal violet solution (in dH2O). Cells were washed with dH2O and air dried overnight. Then 1% Triton-X solution (in PBS) was added, and the plate gently agitated for 15 min. The solution was then transferred to a 96-well plate, read at 590 nm on a spectrophotometer and plotted to determine relative cell numbers.

### 4.4. Characterizing the Effect of Tuberculosis Antimicrobials on Metabolism Profiles and Mitochondrial Function Utilizing the Seahorse XFe24 Analyzer

After 7–10 days of culturing and differentiating, hMDMs were scraped, counted, and re-seeded at 2 × 10^5^ cells per well in a 24-well cell culture XF microplate (Seahorse Biosciences, North Billerica, MA, USA), incubated for 24 h, washed with complete RPMI, infected with iH37Rv-Mtb, and treated with tuberculosis antimicrobials as described above. hMDMs were then incubated for a further 24 h, washed with assay medium (Seahorse XF medium, supplemented with 14.6 mg L-glutamine, 500 µL glucose, and 500 µL pyruvate) (Agilent, Santa Clara, CA, USA) before incubation with assay medium for 1 h at 37 °C in a non-CO_2_ incubator. A mitochondrial stress test was carried out, according to the manufacturer’s instructions.

OCR and ECAR, reflecting OXPHOS and glycolysis, respectively, were measured before and after treatment with oligomycin (1 μM), FCCP (1 μM) and antimycin-A/rotenone (0.5 μM) (XF Cell Mito Stress Kit, Biosciences) using the Seahorse XF*_e_*24 analyzer (Seahorse Biosciences). Three baseline OCR and ECAR measurements were obtained over 20 min prior to injection of oligomycin, FCCP, and antimycin-A/rotentone. Three subsequent OCR and ECAR measurements were also obtained over 15 min following injection with oligomycin, FCCP, and antimycin-A/rotenone. Non-mitochondrial respiration was calculated by expressing residual OCR post antimycin-A/rotenone injection as a percentage of baseline OCR. Spare respiratory capacity was calculated by plotting the percentage change in OCR post FCCP injection versus baseline OCR. Mitochondrial coupling efficiency was calculated by plotting the percentage change in ATP production rate versus baseline OCR. Proton leak was calculated by plotting percentage change in OCR post oligomycin injection versus baseline OCR post antimycin-A/rotenone addition. The experiment was repeated a minimum of 5 times (*n* = 5–10), with at least two technical replicates. All measurements were normalized to cell number using the crystal violet assay.

### 4.5. Ex Vivo MSD Multiplex ELISA Analysis

hMDMs were differentiated and adherence purified from PBMCs, stimulated with iH37Rv, and treated with antimicrobials as well as appropriate controls, as described above. Supernatants collected at 24 h post infection were screened for the levels of cytokines/chemokines according to manufacturers’ instructions (Meso Scale Discovery Multi-Array Technology, Rockville, MD, USA). The cytokines assessed included IL-1β, IL-6, IL-8, IL-10, TNFα, IFN-γ, IL-12, IL-13, IL-2, and IL-4. The chemokines assessed included MCP-1, MCP-4, IP-10, MDC, MIP-1β, Eotaxin, Eotaxin-3, TARC, and MIP-1α.

### 4.6. Statistical Analysis

Data were analysed using GraphPad Prism software version 9 (GraphPad Prism, San Diego, CA, USA). Two-way repeated measures ANOVA tests with Šídák’s multiple comparisons were utilised to statistically analyse differences in OCR, ECAR, OCR:ECAR, spare respiratory capacity, non-mitochondrial respiration, mitochondrial coupling efficiency, and mitochondrial proton leak. Meso Scale Discovery Multi-Array technology chemokine and cytokine assays were statistically analysed using two-way repeated measures ANOVA tests with Šídák’s multiple comparisons tests. Differences of *p* < 0.05 (*), *p* < 0.01 (**), *p* < 0.001 (***), and *p* < 0.0001 (****) were considered statistically significant.

## Figures and Tables

**Table 1:** Overview of TB drugs (mechanism of action and known effects on eukaryotic cells).

Drug Name	Abbreviation	Clinical Use	In Vitro Solvent	Mechanism of Action	Known Effects on Eukaryotic Cells
Ethambutol	EMB	First-line	dH_2_O	Functions by obstructing the formation of cell wall	Inhibits complex IV and OXPHOS in human fibroblasts [12]
Isoniazid	INH	First-line	dH_2_O	Inhibits mycolic acid synthesis; interferes with cell wall synthesis	Reduces complex I-III activity & increases ROS and lipid peroxidation in rat mitochondria [13] and alters murine mitochondria [14]
Pyrazinamide	PYZ	First-line	dH_2_O	Interferes with the bacteriums’ ability to synthesize new fatty acids (required for growth and replication)	Associated with increased ROS production in zebrafish larvae [15]
Rifampicin	RIF	First-line	dH_2_O	Inhibits bacterial DNA-dependent RNA synthesis	Alters structure of mitochondria resulting in excessive ROS and release of cyto-chrome c [16] and alters murine mitpochondria [14]
Amikacin	AMK	Second-line	dH_2_O	Interferes with mRNA binding (interferes with bacterial growth)	Unknown effects on eukaryotic cells [9]
Bedaquiline	BDQ	Second-line	DMSO	Inhibits the proton pump of mycobacterial ATP synthase (essential for ATP production)	Inhibits the mitochondrial transport chain in rat models [17], reduces mitochondrial membrane potential and increases ROS production in human breast cancer cells [18]
Clofazimine	CLO	Second-line	DMSO	Blocks the template function of DNA, inhibiting proliferation	CLO increases glycolysis in human non-small-cell bronchial-carcinoma cells [19]
Cycloserine	CYS	Second-line	dH_2_O	Inhibits cell-wall biosynthesis	Unknown effects on eukaryotic cells [9]
Linezolid	LIN	Second-line	DMSO	Inhibits bacterial protein synthesis	Decreases protein levels and the enzymatic activity of various mitochondrial complexes [20]
Moxifloxacin	MOX	Second-line	dH_2_O	Blocks bacterial DNA replication	Exhibits anti-inflammatory effects in human THP-1 cells [21]

## Data Availability

See Supplementary Material for all raw data in this study.

## Supplementary Materials

Supplementary materials can be found at https://www.mdpi.com/article/10.3390/ijms222212189/s1.
