# Supplementary material for: The Effect of Tuberculosis Antimicrobials on the Immunometabolic Profiles of Primary Human Macrophages Stimulated with Mycobacterium tuberculosis
Source: Int J Mol Sci. 2021 Nov 10;22(22):12189. doi: 10.3390/ijms222212189 (PMC8624646; doi:10.3390/ijms222212189)
Supplement: Supplementary file 1 [file ijms-22-12189-s001.zip › final supps/ijms-1342319 supp figures.docx]

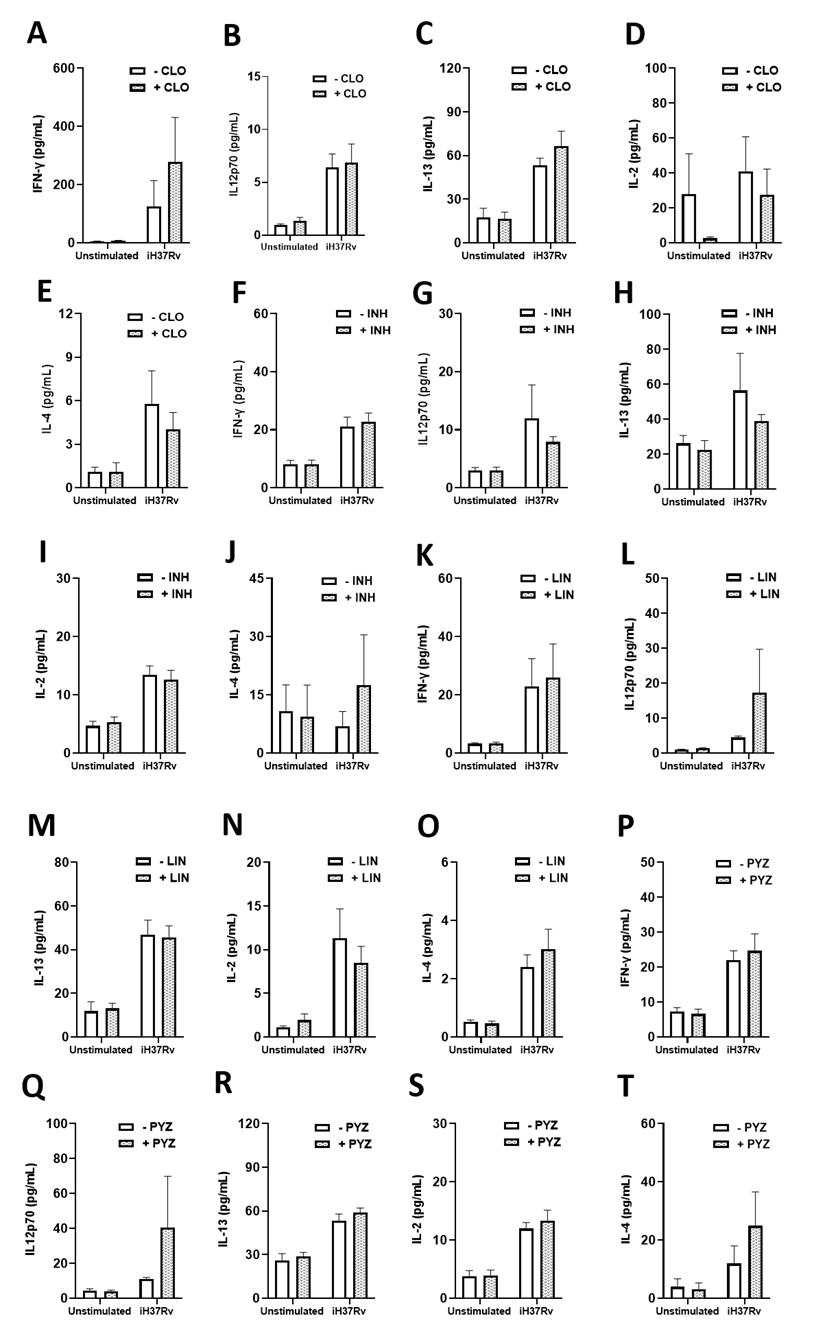


**Supplementary** **Figure 1.** Examining the effect of clofazimine, isoniazid, linezolid and pyrazinamide on protein levels of IFN-γ, IL-12, IL-13, IL-2 and IL-4 in hMDMs stimulated with iH37Rv-Mtb. hMDMs, differentiated from PBMCs isolated from healthy blood donors, were stimulated with iH37Rv-Mtb for 3 h, washed to remove unphagocytosed Mtb, and were treated with clofazimine (2 µg/mL), isoniazid (1 µg/mL), linezolid (15 µg/mL) or pyrazinamide (2 µg/mL). 24 h post stimulation, protein levels of IFN-γ (A,F, K and P), IL-12 (B, G, L and Q), IL-13 (C, H, M and R), IL-2 (D, I, N and S) and IL-4 (E, J, O and T) were quantified using Meso Scale Discovery Multi-Array technology. Bars denote mean ± SEM. **p* < 0.05 (Two-way repeated measures ANOVA tests with Šídák’s multiple comparisons tests).


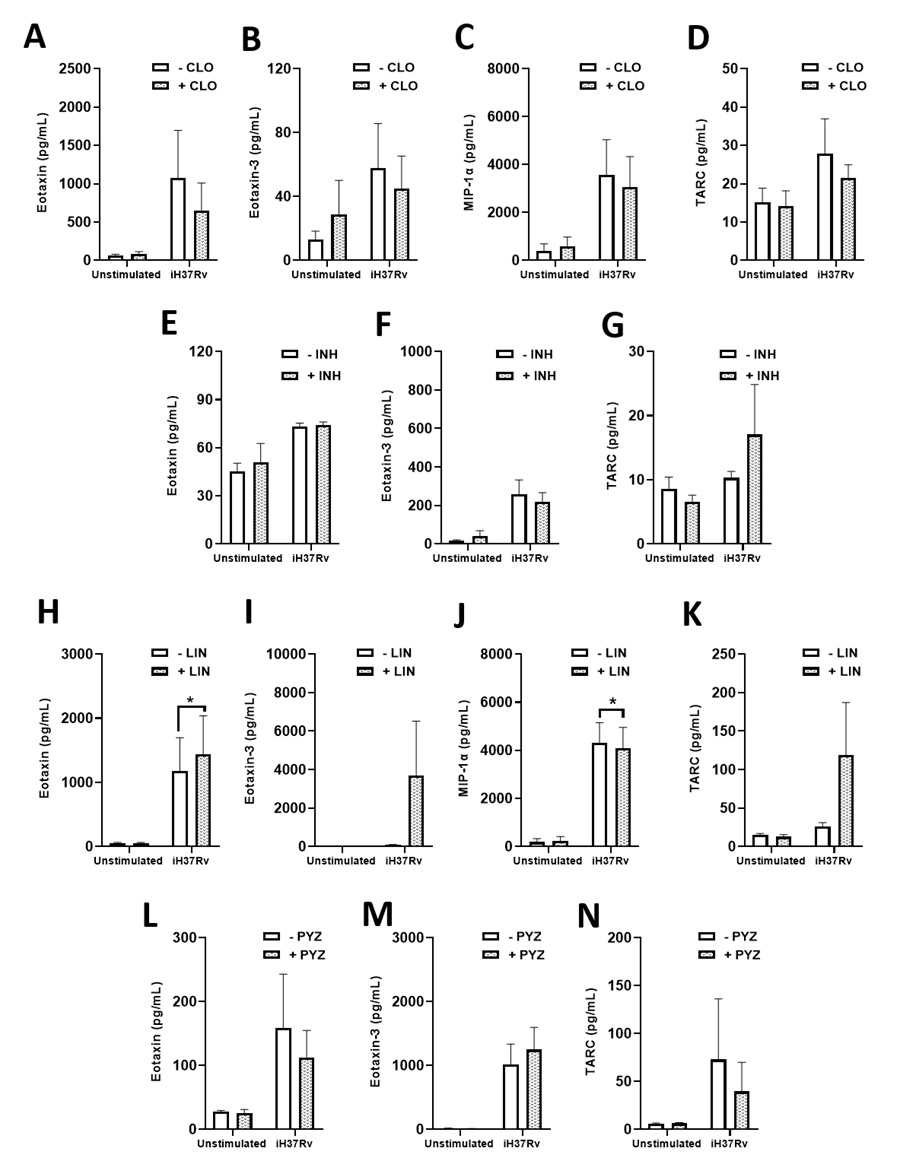


**Supplementary** **Figure** **2.** Examining the effect of clofazimine, isoniazid, linezolid and pyrazinamide on protein levels of Eotaxin, Eotaxin-3, TARC and MIP-1α in hMDMs stimulated with iH37Rv-Mtb. hMDMs, differentiated from PBMCs isolated from healthy blood donors, were stimulated with iH37Rv-Mtb for 3 h*,* washed to remove unphagocytosed Mtb*,* and were treated with clofazimine (2 µg/mL), isoniazid (1 µg/mL), linezolid (15 µg/mL) or pyrazinamide (2 µg/mL). 24 h post stimulation, protein levels of Eotaxin (**A**,**E**,**H** and **L**), Eotaxin-3 (**B**,**F**,**I** and **M**), MIP-1α (**C** and **J**), and TARC (**D**,**G**,**K** and **N**) were quantified using Meso Scale Discovery Multi-Array technology. Bars denote mean ± SEM. **p* < 0.05 (Two-way repeated measures ANOVA tests with Šídák’s multiple comparisons tests).


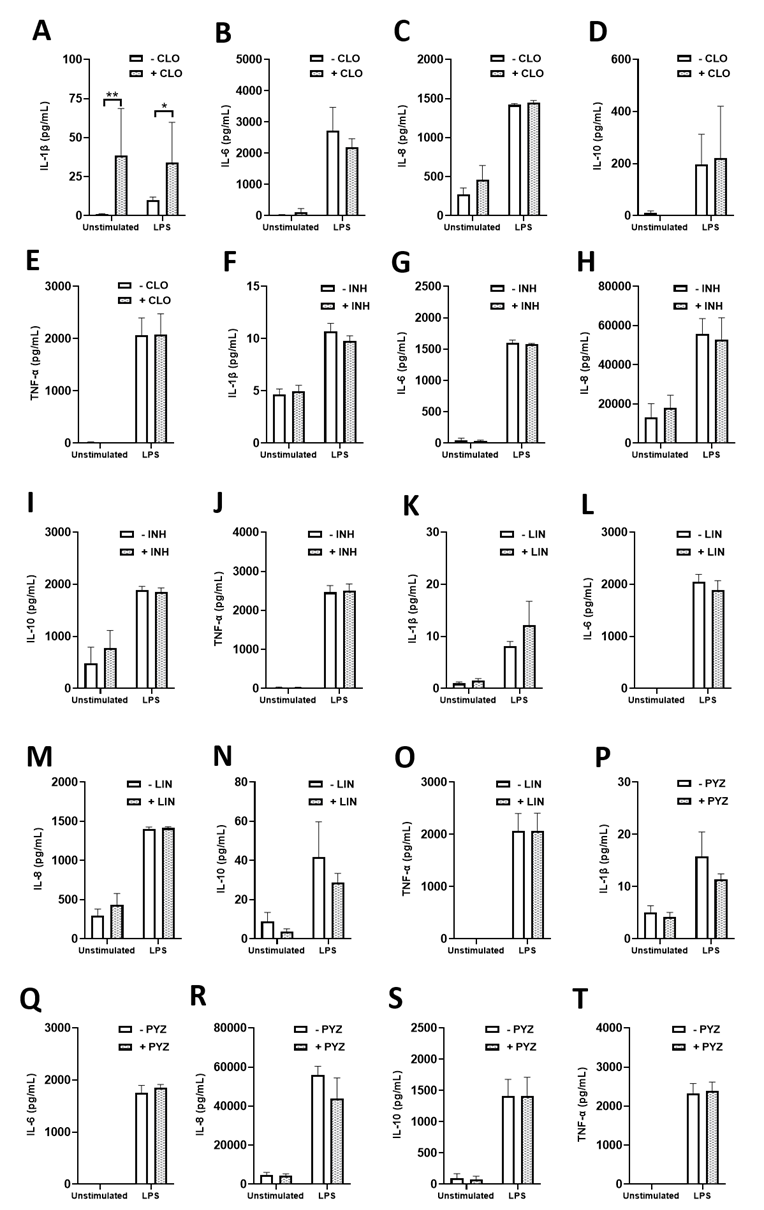


**Supplementary** **Figure** **3.** Examining the effect of clofazimine, isoniazid, linezolid and pyrazinamide on protein levels of IL-1β, IL-6, IL-8, IL-10 and TNFα in hMDMs stimulated with LPS. hMDMs, differentiated from PBMCs isolated from healthy blood donors, were stimulated with LPS (100 ng/mL) and were treated with clofazimine (2 µg/mL), isoniazid (1 µg/mL), linezolid (15 µg/mL) or pyrazinamide (2 µg/mL) 3 h post stimulation. 24 h post LPS stimulation, protein levels of IL1β (**A**,**F**,**K** and **P**), IL-6 (**B**,**G**,**L** and **Q**), IL-8 (**C**,**H**,**M** and **R**), IL-10 (**D**,**I**,**N** and **S**) and TNFα (**E**,**J**,**O** and **T**) were quantified using Meso Scale Discovery Multi-Array technology. Bars denote mean ± SEM. **p* < 0.05 (Two-way repeated measures ANOVA tests with Šídák’s multiple comparisons tests).


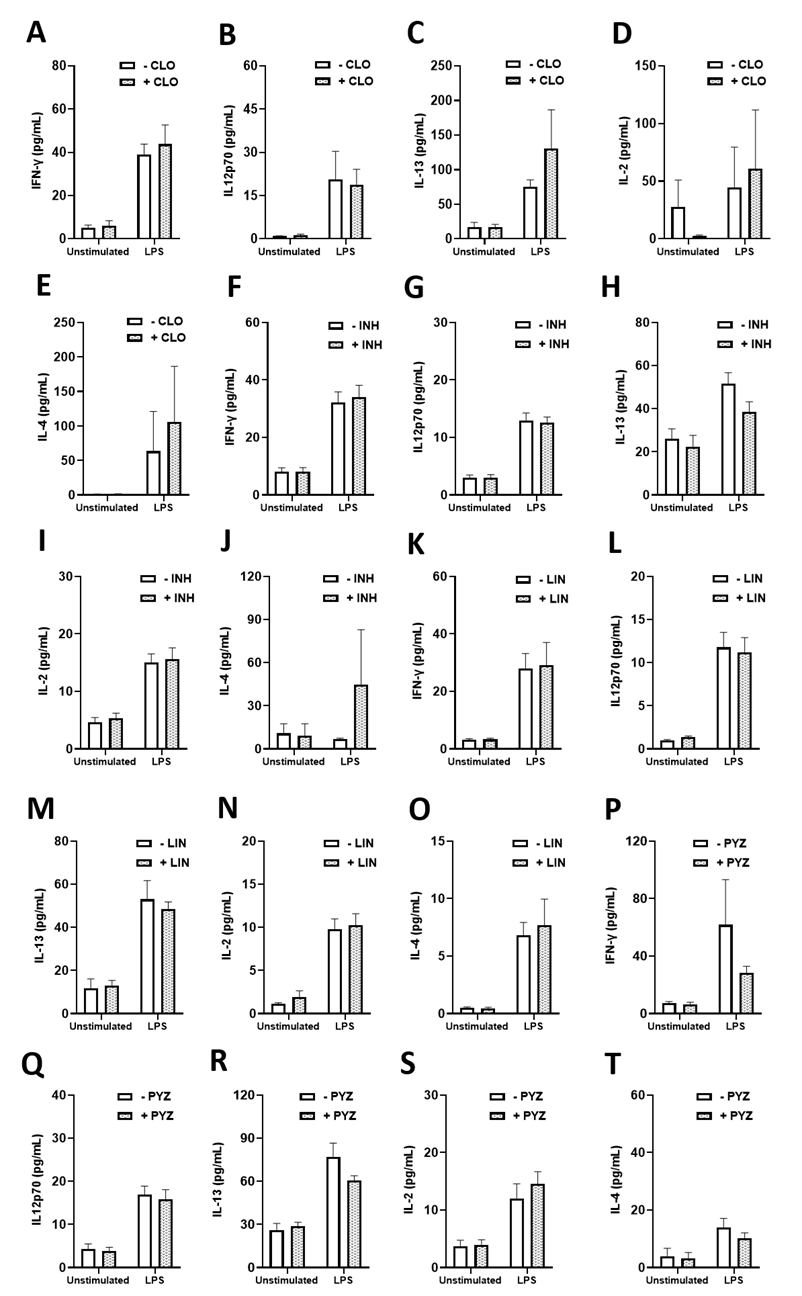


**Supplementary** **Figure** **4.** Examining the effect of clofazimine, isoniazid, linezolid and pyrazinamide on protein levels of IFN-γ, IL-12, IL-13, IL-2 and IL-4 in hMDMs stimulated with LPS. hMDMs, differentiated from PBMCs isolated from healthy blood donors, were stimulated with LPS (100 ng/mL) and were treated with clofazimine (2 µg/mL), isoniazid (1 µg/mL), linezolid (15 µg/mL) or pyrazinamide (2 µg/mL) 3 h post stimulation. 24 h post LPS stimulation, protein levels of IFN-γ (**A**,**F**,**K** and **P**), IL-12 (**B**,**G**,**L** and **Q**), IL-13 (**C**,**H**,**M** and **R**), IL-2 (**D**,**I**,**N** and **S**) and IL-4 (**E**,**J**,**O** and **T**) were quantified using Meso Scale Discovery Multi-Array technology. Bars denote mean ± SEM. **p* < 0.05 (Two-way repeated measures ANOVA tests with Šídák’s multiple comparisons tests).


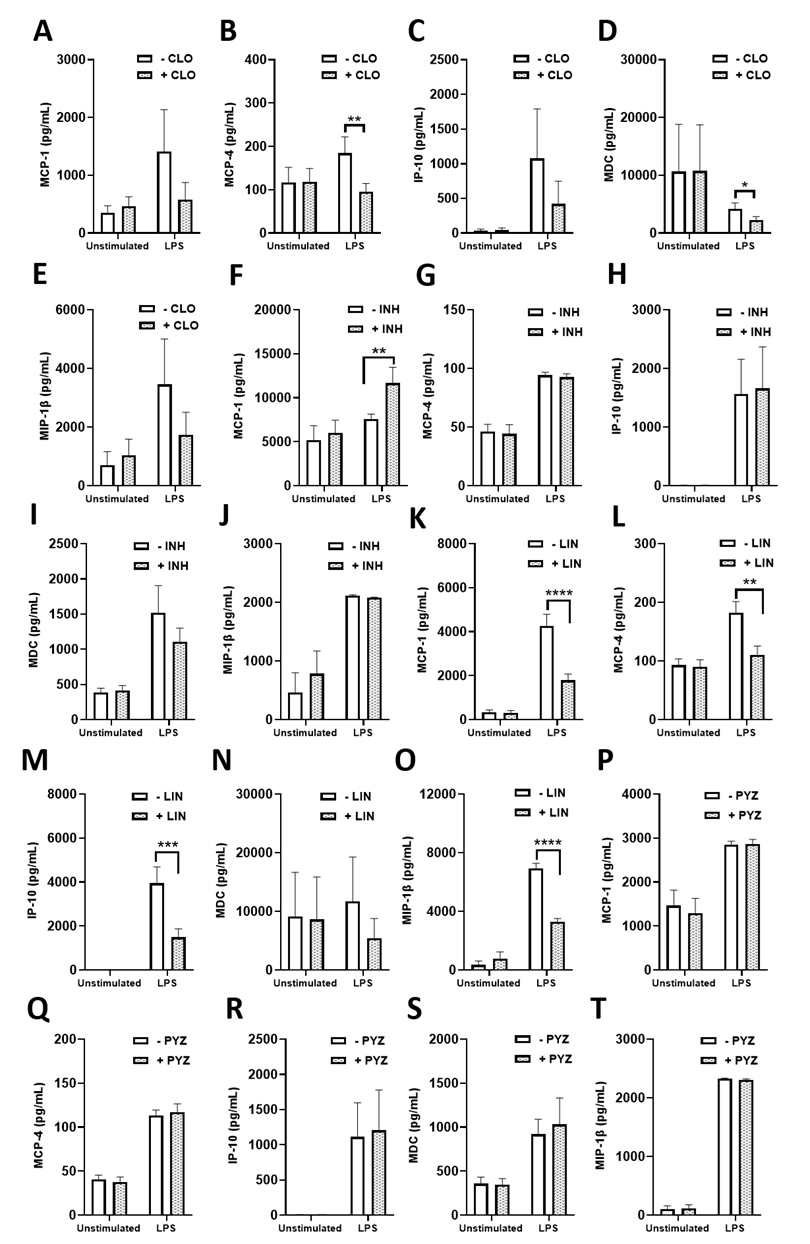


**Supplementary** **Figure** **5.** Assessing the effect of clofazimine, isoniazid, linezolid and pyrazinamide on protein levels of MCP-1, MCP-4, IP-10, MDC and MIP-1β in hMDMs stimulated with LPS. hMDMs, differentiated from PBMCs isolated from healthy blood donors, were stimulated with LPS (100 ng/mL) and were treated with clofazimine (2 µg/mL), isoniazid (1 µg/mL), linezolid (15 µg/mL) or pyrazinamide (2 µg/mL) 3 h post stimulation. 24 h post stimulation, protein levels of MCP-1 (**A**,**F**,**K** and **P**), MCP-4 (**B**,**G**,**L** and **Q**), IP-10 (**C**,**H**,**M** and **R**), MDC (**D**,**I**,**N** and **S**) and MIP-1β (**E**,**J**,**O** and **T**) were quantified using Meso Scale Discovery Multi-Array technology. Bars denote mean ± SEM. **p* < 0.05 (Two-way repeated measures ANOVA tests with Šídák’s multiple comparisons tests).


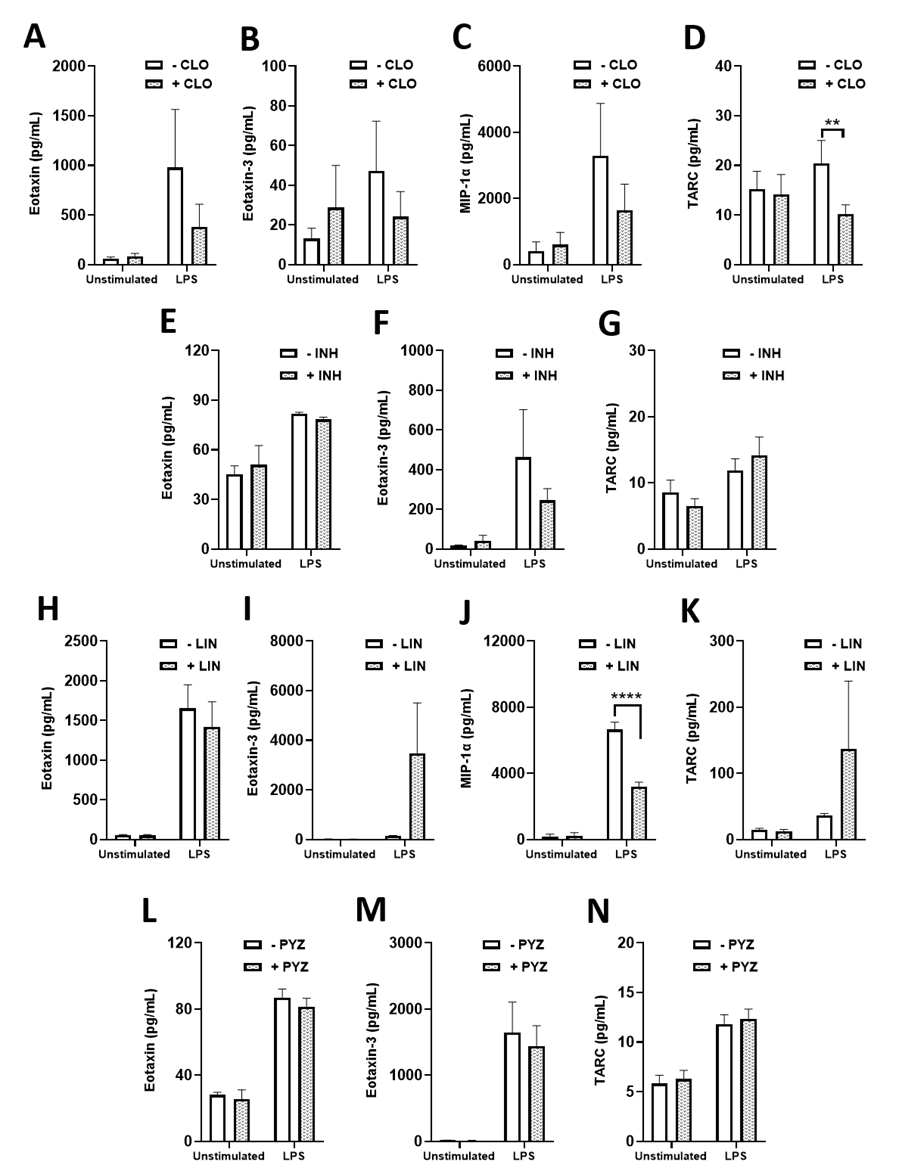


**Supplementary** **Figure** **6.** Examining the effect of clofazimine, isoniazid, linezolid and pyrazinamide on protein levels of Eotaxin, Eotaxin-3, TARC and MIP-1α in hMDMs stimulated with LPS. hMDMs, differentiated from PBMCs isolated from healthy blood donors, were stimulated with LPS and were treated with clofazimine (2 µg/mL), isoniazid (1 µg/mL), linezolid (15 µg/mL) or pyrazinamide (2 µg/mL) 3 h post stimulation. 24 h post stimulation, protein levels of Eotaxin (**A**,**E**,**H** and **L**), Eotaxin-3 (**B**,**F**,**I** and **M**), MIP-1α (**C** and **J**), and TARC (**D**,**G**,**K** and **N**) were quantified using Meso Scale Discovery Multi-Array technology. Bars denote mean ± SEM. * *p* < 0.05 (Two-way repeated measures ANOVA tests with Šídák’s multiple comparisons tests).
